# Effects of Eleutheroside B and Eleutheroside E on activity of cytochrome P450 in rat liver microsomes

**DOI:** 10.1186/1472-6882-14-1

**Published:** 2014-01-02

**Authors:** Sixun Guo, Yan Liu, Zhiping Lin, Sheng Tai, Shuo Yin, Gaofeng Liu

**Affiliations:** 1 Department of Pharmacy, the Second Affiliated Hospital of Harbin Medical University, Harbin 150086, China; 2 Department of General Surgery, the Second Affiliated Hospital of Harbin Medical University, Harbin 150086, China

**Keywords:** Eleutheroside B, Eleutheroside E, Cytochrome P450, In vitro

## Abstract

**Background:**

Chemicals of herbal products may cause unexpected toxicity or adverse effect by the potential for alteration of the activity of CYP450 when co-administered with other drugs. *Eleutherococcus senticosus* (ES), has been widely used as a traditional herbal medicine and popular herbal dietary supplements, and often co-administered with many other drugs. The main bioactive constituents of ES were considered to be eleutherosides including eleutheroside B (EB) and eleutheroside E (EE). This study was to investigate the effects of EB and EE on CYP2C9, CYP2D6, CYP2E1 and CYP3A4 in rat liver microsomes *in vitro*.

**Method:**

Probe drugs of tolbutamide (TB), dextromethorphan (DM), chlorzoxazone (CLZ) and testosterone (TS) as well as eleutherosides of different concentrations were added to incubation systems of rat liver microsomes *in vitro*. After incubation, validated HPLC methods were used to quantify relevant metabolites.

**Results:**

The results suggested that EB and EE exhibited weak inhibition against the activity of CYP2C9 and CYP2E1, but no effects on CYP2D6 and CYP3A4 activity. The IC_50_ values for EB and EE were calculated to be 193.20 μM and 188.36 μM for CYP2E1, 595.66 μM and 261.82 μM for CYP2C9, respectively. Kinetic analysis showed that inhibitions of CYP2E1 by EB and EE were best fit to mixed-type with Ki value of 183.95 μM and 171.63 μM, respectively.

**Conclusions:**

These results indicate that EB and EE may inhibit the metabolism of drugs metabolized via CYP2C9 and CYP2E1, and have the potential to increase the toxicity of the drugs.

## Background

**Figure 1:**
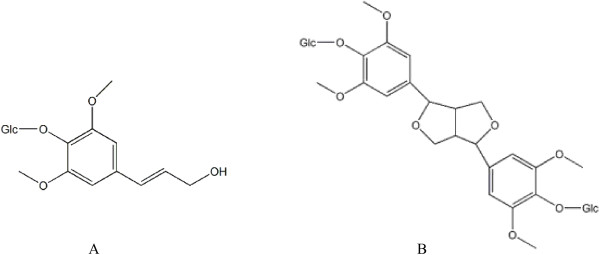
**Chemical structures of eleutheroside B and eleutheroside E.** Glc are connected by β-D-glycosidic linkages. **(A)** Eleutheroside B **(B)** Eleutheroside E

*Eleutherococcus senticosus* (ES, *Acanthopanax senticosus*), also called *Siberian ginseng* in the Siberian Taiga region and *Ciwujia* in China, belongs to the family of *Araliaceae*, are mainly distributed in the far-eastern region of Russia, the northeastern of China, Japan and Korea. It is a medicinal herb that dates back more than 2000 years according to Chinese medicine records and is also known as a powerful tonic herb with an impressive range of health benefits. This medicinal plant is not only popular in China and Russia, but also one of the 10 popular herbal dietary supplements used in the United States [1]. Recently, ES has drawn increasingly attention due to its excellent effects on invigorating spleen, benifitting liver and nourishing kidney [2], and lots of chemical, pharmacological and clinical studies on ES have been carried out all over the world [3,4]. The main bioactive constituents of ES were considered to be eleutherosides including eleutheroside B (EB) and eleutheroside E (EE) (Figure 1). As the quality standard of ES, the amount of EB and EE should be more than 0.8% according to the “United States pharmacopoeia” and “European pharmacopoeia” [5]. EB possessed anti-stress, anti-fatigue [6], anti-oxidant [7], anti-irradiation, anti-gastric ulceration, anti-inflammatory [8], immunopotentiating [9], immunomodulatory [10], anti-diabetic effects [11,12] etc. Besides, EB and EE showed obvious protective effects against neuritic atrophy and nerve cell death [13,14]. Furthermore, EE exerted significant anti-inflammatory effects by suppressing the gene expression of inflammatory proteins and protective effects in ischemia heart [15]. In addition, EE has the potential abilities to alleviate behavioral alterations induced by sleep deprivation [16] and fatigue both in physical and mental fatigue [17].

Cytochrome P450 (CYP450), the most important drug-metabolizing enzymes, with largest number and highest abundance of CYP isoforms present in the liver. It is well known that CYP450 play a vital role in the metabolism of currently used drugs. Most drugs are metabolized through several major CYP isoforms. CYP3A4, CYP2D6, CYP2C9 and CYP2E1 have been estimated to be involved in the metabolism of approximately 85% of the drugs in clinical practice [18]. Since these subtypes metabolize numerous drugs, drug-drug interactions (DDIs) can potentially occur when multiple drugs are co-administered. Therefore, evaluation of effects on CYP isoforms of drugs is important for the prediction of potential DDIs. Inhibition of CYP450 enzymes is the most important reason caused DDIs, which raises the toxicity of drugs.

To our knowledge, this is the first study focus on the potential role of EB and EE in CYP450-mediated DDIs. The present study was performed to investigate the influence of EB and EE on the activities of CYP2C9, CYP2D6, CYP2E1 and CYP3A4 in rat liver microsomes and the mechanisms *in vitro*, which could be used to predict the probability of herb-drug interactions, providing references for correlation studies and decrease the risk of drug use.

## Methods

### Chemicals and reagents

Tolbutamide was purchased from Dr. Ehrenstorfer GmbH (Augsburg, Germany). 4-hydroxytolbutamide and 6-hydroxychlorzoxazone were obtained from Toronto Research Chemicals Inc. (North York, Canada). Dextromethorphan, dextrorphan and chlorzoxazone were supplied by Sigma-Aldrich Co. (St Louis, MO, USA). Testosterone was obtained from International Laboratory Limited (San Bruno, CA, USA). 6β-hydroxytestosterone was purchased from BD Biosciences Co. (Woburn, MA, USA). Phenacetin, cortisone acetate, EB and EE were from National Institute for the Control of Pharmaceutical and Biological Products (Beijing, China). NADPH was obtained from Roche Diagnostics GmbH (Mannheim, Germany). All other reagents were of HPLC or analytical grade.

### Preparation of rat liver microsomes

Wistar rats (180 ± 20 g, male) were supplied by the Animal Experimental Center of Harbin Medical University (Harbin, China), which was fully accredited by the Guide for the Care and Use of Laboratory Animals of the National Institutes of Health. The protocol was approved by the Committee on the Ethics of the Harbin Medical University (Permit Number: HMUIRB20120011), and the rats were handled in a manner that met all the recommendations formulated by the National Society for Medical Research and Guidelines for the Care and Use of Laboratory Animals. Rat liver microsomes were prepared from liver tissue by differential ultra-centrifugation [19], packed and stored at -80°C for further analysis. These operations were carried out in the ice. Protein concentrations of the microsomes were determined by the method of Bradford [20].

### Cytochrome P450 probe substrate assays

#### Tolbutamide and 4-methyhydroxylation assay for CYP2C9

The incubation system of CYP2C9 *in vitro* contained phosphate buffer (100 mM_,_ pH7.4), liver microsomal protein (0.5 mg · mL^-1^), MgCl_2_ (10 mM), tolbutamide (90 μM) and eleutherosides in a final volume of 200 μL. Pre-incubated 5 min, the reaction was initiated by adding NADPH (1 mM concentration in incubation) and the incubation systems were incubated at 37°C for 60 min. After incubation, 50 μL ice-cold acetonitrile was added to terminate the reaction, and phenacetin of a final concentration 20 μM was added as internal standard. With 5 min suspension, the mixture was centrifuged for 30 min at 12000 r · min^-1^. The supernatant of 20 μL was analyzed by the Waters HPLC system 2010 (Waters, USA, with 600 pump, 996PAD UV detector and Millipore Systems). Tolbutamide, 4-hydroxytolbutamide and phenacetin were separated on a Diamonsil C_18_ reverse phase column (5 μm, 4.6 mm × 200 mm). The column temperature was set to 35°C. The mobile phase, at a flow rate of 1 mL · min^-1^, consisted of methanol and 0.1% acetic acid (55:45, v/v). UV detection was at wavelength of 229 nm. The organic solvent which is at low concentration (≤0.5%) in all incubation systems wouldn’t affect the activity of enzymes. The yield of corresponding metabolites was calculated by referring to a standard curve constructed based on known concentrations of the pure metabolites.

#### Dextromethorphan and O-demethylation assay CYP2D6

Incubation conditions were the same as Section Tolbutamide and 4-methyhydroxylation assay for CYP2C9. The liver microsomal protein was 1.0 mg · mL^-1^ and tolbutamide was replaced by 25 μM dextromethorphan. Reactions were terminated by 80 μL ice-cold acetonitrile and internal standard phenacetin (final concentration of 50 μM) was added, the denatured protein was removed by centrifuged at 12000 r · min^-1^ for 30 min. The supernatant of 20 μL was injected into the HPLC system, with the mobile phase of methanol, water, phosphate and triethylamine (42:58:0.15:0.3, v/v/v/v) at a flow rate of 1 mL · min^-1^, detection was at wavelength of 280 nm.

#### Chlorzoxazone and 6-hydroxylation assay for CYP2E1

Each incubation mixture (200 μL) included liver microsomal protein (0.75 mg. mL^-1^), MgCl_2_ (10 mM) in 100 mM phosphate buffer (pH7.4) and 25 μM chlorzoxazone. With 5 min pre-incubation, all reactions were initiated by addition of NADPH (1 mM) and were carried out in 37°C water bath for 30 min, and then were stopped by addition of 150 μL ice-cold acetonitrile and internal standard (80 μM phenacetin). After centrifugation at 12000 r · min^-1^ for 30 min, 20 μL of the supernatant was injected into the HPLC system, and eluted with methanol–water (47:53) at a flow rate of 1.0 mL · min^-1^, UV absorbance was monitored at 287 nm.

#### Testosterone and 6β-hydroxylation assay for CYP3A4

Testosterone solution (in methanol, final concentration of 100 μM) was evaporated to dryness under nitrogen in 40°C water bath, then additional reagents were added to give a final incubation volume of 200 μL: liver microsomal protein (0.5 mg · mL^-1^) in 50 mM sodium phosphate buffer (pH7.4) and MgCl_2_ (10 mM). Following a 5 min pre-incubation, reactions were started with addition of NADPH (1 mM). Following 30 min incubations at 37°C, reactions were stopped with organic solution (280 μL ice-cold acetonitrile), and cortisone acetate was added as internal standard with final concentration of 12.5 μM. The mixture was centrifugated at 12000 r · min^-1^ for 30 min, and the supernatant of 20 μL was injected into the HPLC, with UV detection at 245 nm. Mobile phase consisted of methanol and water (65:35, v/v), and the flow rate was 1.0 mL · min^-1^.

### Determination of K_m_ and V_max_

The apparent K_m_ (Michaelis constant) and V_max_ (maximum reaction velocity) values were determined in a range of concentrations of probe drugs. The concentrations were as follows: tolbutamide 3.5~600.0 μM, dextromethorphan 3.5~400.0 μM, chlorzoxazone 5.0~300.0 μM, and testosterone 12.5~500.0 μM. The other incubation conditions were the same as Section Cytochrome P450 probe substrate assays.

### Determination of effects of EB and EE on CYP450 activity

To evaluate whether EB and EE affect the activity of CYP450, the probe substrate reaction assays were performed with EB or EE at concentrations of 0, 2, 10, 25, 50, 150, 300 μM under the conditions described earlier, with triplicate incubations for each concentration. The concentrations of respective probe substrates were selected according to K_m_ established in the enzyme kinetic assays described above. The IC_50_ values (concentration of inhibitor causing 50% inhibition of enzyme activity) were determined based on the concentration-inhibition curves.

### Assay for enzymatic kinetic parameters

The exact inhibition constants (K_i_ values) were measured and the modes of inhibition were determined for the components exhibiting IC_50_ values of less than 200 μM. The K_i_ values were determined in a range of concentrations of probe substrates (approximately K_m_/2, K_m,_ 2K_m_ and 4K_m_) and different concentrations of EB (0, 100, 200, 300 μM) and EE(0, 100, 200, 300 μM). Dixon and Lineweaver-Burk plots and the second plots showed the data graphically for interpretation of inhibition mode. All experiments were separately performed in three times.

## Results

### Enzymatic kinetic parameters for CYP450 in rat liver microsomes

The apparent K_m_ values for tolbutamide 4-methyhydroxylation, dextro- methorphan O-demethylation, chlorzoxazone 6-hydroxylation and testosterone 6β-hydroxylation in rat liver microsomes were 94.80, 29.07, 30.42 and 103.81 μM, respectively. The V_max_ values for tolbutamide 4-methyhydroxylation, dextromethorphan O-demethylation, chlorzoxazone 6-hydroxylation and testosterone 6β-hydroxylation in liver microsomes were 1.57, 0.28, 0.05 and 2.71 nmol/min/mg protein, respectively.

### IC_50_ for EB and EE inhibition

**Figure 2:**
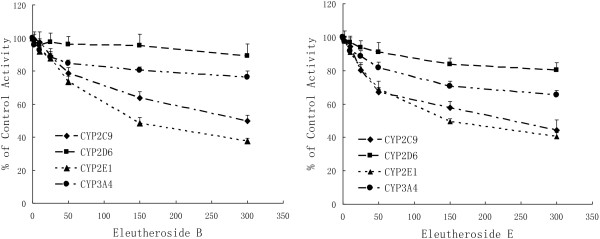
Concentration-inhibition curves of CYP enzymes by EB and EE (n = 3).

The inhibitory potency of EB and EE were determined based by the concentration-inhibition curves of the four CYP isoforms. As shown in Figure 2, EB inhibited tolbutamide 4-methyhydroxylation (CYP2C9) with an IC_50_ of 595.66 μM and chlorzoxazone 6-hydroxylation (CYP2E1) with an IC_50_ of 193.20 μM. The IC_50_ values of EE were 261.82 μM for CYP2C9 and 188.36 μM for CYP2E1 (Figure 2), respectively. The results indicated that EB and EE can inhibit CYP2E1 and CYP2C9 activities in rat liver microsomes. On the other hand, EB and EE did not cause inhibition of CYP2D6, CYP3A4, the IC_50_ values could not be extrapolated and calculated.

### Type of inhibition of CYP2E1 by EB and EE

**Figure 3:**
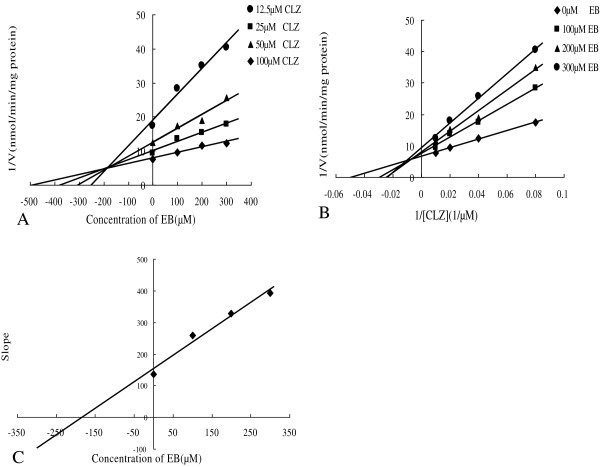
**Kinetic study of the effect of EB on CYP2E1 enzyme (A)** Dixon plot of inhibition effect of EB on chlorzoxazone 6-hydroxylation (CYP2E1). **(B)** Lineweaver-Burk plot of inhibitory effect of EB on chlorzoxazone 6-hydroxylation (CYP2E1). **(C)** Secondary plot of the slopes from Lineweaver-Burk plot vs EB concentrations. Each data point represents mean of triplicate incubations.

**Figure 4:**
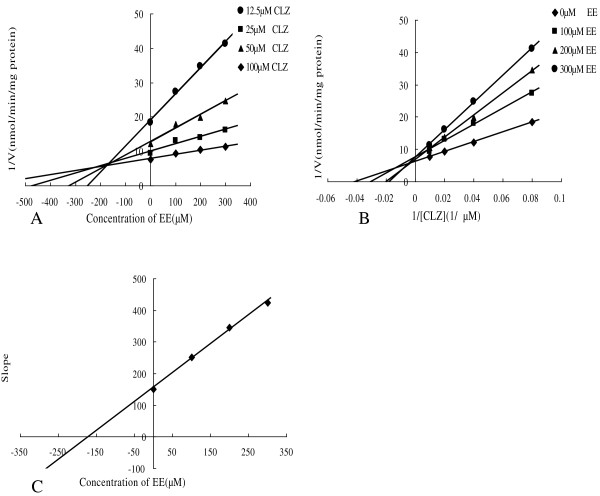
**Kinetic study of the effect of EE on CYP2E1 enzyme. (A)** Dixon plot of inhibition effect of EE on chlorzoxazone 6-hydroxylation (CYP2E1). **(B)** Lineweaver-Burk plot of inhibitory effect of EE on chlorzoxazone 6-hydroxylation (CYP2E1). **(C)** Secondary plot of slopes from Lineweaver-Burk plot versus EB concentrations. Each data point represents mean of triplicate incubations.

To further characterize the inhibition of CYP2E1 activity by EB and EE, K_i_ values were calculated and the inhibition type were determined with EB and EE concentrations of 0, 100, 200, 300 μM. Dixon and Lineweaver-Burk plots showed that the inhibition of EB to CYP2E1 was best fit to a mixed-type, and the K_i_ value was evaluated to be 183.95 μM from the secondary plot of the slopes of Lineweaver-Burk plots versus the concentrations of EB (Figure 3). Furthermore, kinetic study of the effect of EE on CYP2E1 enzyme showed that the inhibition was mixed-type with K_i_ of 171.63 μM (Figure 4).

## Discussion

The incubation experiment of liver microsomes is a generally used method for drug metabolism *in vitro*, which is recommended by the relevant guidelines of the United States [21]. The liver microsomes consist of a variety of enzymes, and we chose tolbutamide, dextromethorphan, chlorzoxazone and testosterone, which are extensively used as probe substrates for CYP2C9, CYP2D6, CYP2E1 and CYP3A4 respectively in this study [22-25].

The present study addressed that EB and EE exhibited weak inhibition against rat CYP2C9, CYP2E1 activity, and no effect on CYP2D6 and CYP3A4 activity in rat liver microsomes *in vitro*. The IC_50_ values of EE and EB were 193.20 μM and 188.36 μM for CYP2E1, and 595.66 μM and 261.82 μM for CYP2C9, respectively. Kinetic analysis suggested that the inhibition of CYP2E1 by EB was best fit to a mixed-type with K_i_ value of 183.95 μM, while the inhibition of CYP2E1 by EE was also best fit to a mixed manner with K_i_ value of 171.63 μM. Consistently, Donovan *et al* assessed the influence of standardized extracts of ES on the activity of CYP2D6 and CYP3A4 in normal volunteers, and found that there were no statistically significant differences in the pharmacokinetic parameters determined by noncompartmental modeling, indicating that ES are unlikely to significantly affect CYP3A4 and CYP2D6 activities [26].

Our results suggest that EB and EE have inhibition effects on CYP2C9 and CYP2E1 in rat hepatic microsomes. Studies show that the extrapolation of CYP2E1 between species appears to be quite well, and rat seems to be best model for human in this respect [27]. Based on the metabolic substrate studies, human CYP2C9 corresponds to rat CYP2C11, which has quite similar metabolic substrate with human CYP2C9 [28].

CYP2C9 metabolizes more than 10% of drugs clinically used, including antidepressants, hypoglycemics, angiotensin II blockers, non-steroidal anti-inflammatory drugs, S-warfarin, diclofenac, tolbutamide, glipizide, losartan, flurbiprofen, naproxen, ibuprofen, fluoxetine, sertraline, valproate, phenobarbital, phenytoin, fluvastatin, tamoxifen, etc [16,28]. CYP2E1 also plays an important role in the metabolism of many commonly pharmaceuticals including nifedipine, erythromycin, acetaminophen, enflurane, ethanol and halothane [16,28]. The inhibition can result in the increase of the plasma concentration and toxicity of concomitant drugs, especially for those with narrow therapeutic windows such as warfarin, phenobarbital and phenytoin. More attention should be paid when ES is used with these drugs concomitantly. However, further clinical investigation should be carried on to prove whether inhibitions by EB and EE on CYP2C9 or CYP2E1 may alter the pharmacokinetics of these drugs and increase their adverse drug reactions when they are co-administered with ES.

Previous studies have shown that concomitant administration of herbal preparations and pharmaceuticals may affect drug metabolism and significantly increase the risk of serious adverse reactions. For instance, the interaction between warfarin and St. John’s wort have been reported and the mechanism by which St. John’s wort activates CYP450 enzymes is possibly the most thoroughly researched idea [29]. In addition, a number of the clinical relevance of herb interactions has been reported, touching on interactions of *Salvia* (Danshen), *Flos carthami*, *Centella asiatica*, *Andrographis paniculata*, *Silybum marianum*, *Acorus calamus* and *Goldenseal* with various CYP450 isoforms [30-36]. ES has been widely used as a traditional herbal medicine and popular herbal dietary supplements, and often co-administered with many other drugs [37-41], the present study may facilitate predicting possible herb-drug interactions when ES is used in combination with other drugs, and decrease the incidence of the CYP450-mediated interactions.

## Conclusion

In conclusion, EB and EE inhibit CYP2C9 and CYP2E1 activity, but have no effect on CYP2D6 and CYP3A4 in rat liver microsomes *in vitro*. Both compounds show mixed-type inhibition of CYP2C9 and CYP2E1. The results suggest that both EB and EE could cause potential drug-drug interactions with drugs that are metabolized by CYP2C9 and CYP2E1. However, the inhibitory effects of EB and EE on both CYPs were relatively weak and further investigation is needed to evaluate whether the weak inhibitory effects of both compounds on the two CYP450 isoforms are clinically significant.

## Competing interests

Authors of the paper declare that they have no competing interests, and neither financial competing interests nor Non-financial competing interests exist in relation to the manuscript.

## Authors’ contributions

SG and YL carried out the design of the study and the acquisition of HPLC data. ZL and TS participated in the preparation of RLM and the design of relative study. SY participated in statistical analysis. GL was involved in drafting and revising of the manuscript. All authors read and approved the final manuscript.

## Pre-publication history

The pre-publication history for this paper can be accessed here:

http://www.biomedcentral.com/1472-6882/14/1/prepub
